# Supplementary figures and images for: Kar4 is required for the normal pattern of meiotic gene expression
Source: PLoS Genet. 2023 Aug 28;19(8):e1010898. doi: 10.1371/journal.pgen.1010898 (PMC10491391; doi:10.1371/journal.pgen.1010898)

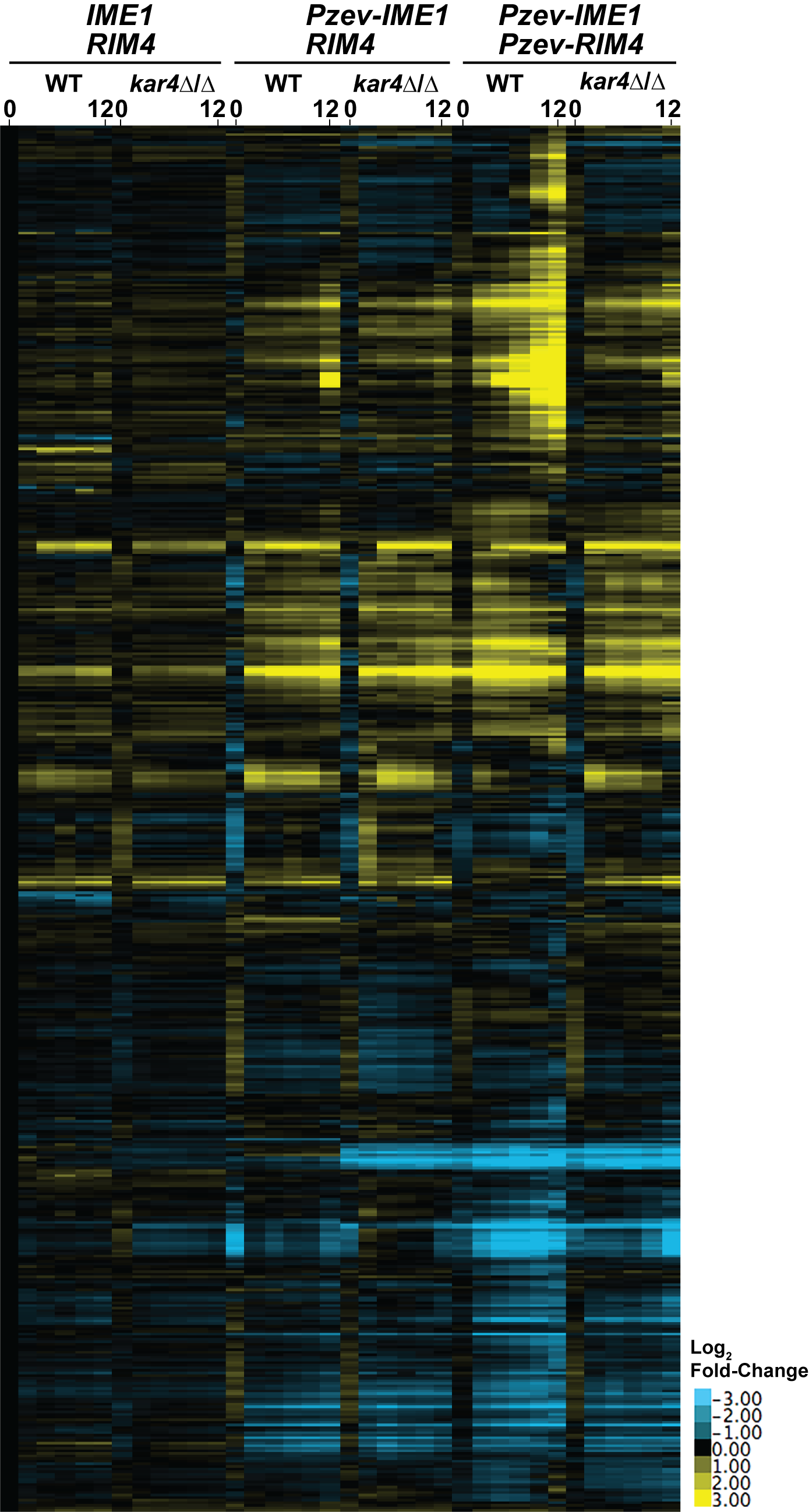

Supplement: S1 Fig — Heatmap of RNA-seq data across a time course of meiosis (0, 2, 4, 6, 8, and 12 hours) in wild type and kar4Δ/Δ with either pIME1/pRIM4, Pzev-IME1/pRIM4, or Pzev-IME1/Pzev-RIM4. Expression was normalized to wild type pre-induction of sporulation (t = 0). Genes were clustered in Cluster3.0 and the heatmaps were constructed with Java TreeView. Note that genes are clustered differently from Fig 1. Source data for heatmap can be found in the supplementary file S3 Data. (TIF) [file pgen.1010898.s001.tif]

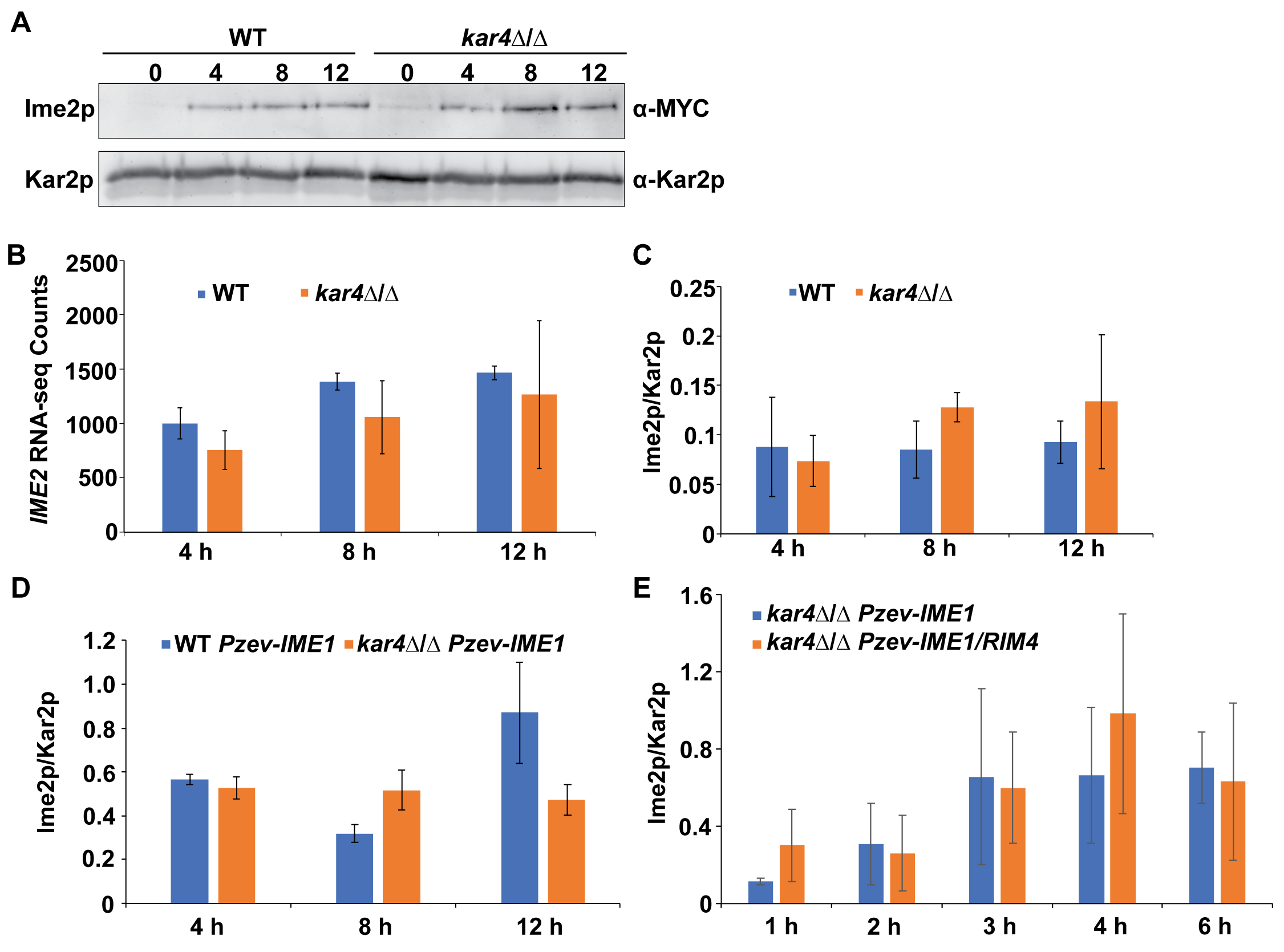

Supplement: S2 Fig — (A) Western blots of Ime2p-13MYC across a meiotic time course in wild type and kar4Δ/Δ. Kar2p is used as a loading control. (B) IME2 RNA-seq normalized counts from wild type and kar4Δ/Δ. Counts were normalized using the standard normalization method in DESeq2. Error bars represent standard deviation between two biological replicates, which is equal to the range divided by the square root of 2. (C) Quantification of western blots in A. Error bars represent the standard deviation between two biological replicates, which is equal to the range divided by the square root of 2. (D) Quantification of western blots in Fig 4A. Error bars represent the standard deviation between two biological replicates, which is equal to the range divided by the square root of 2. (E) Quantification of western blots in Fig 4C. Error bars represent the standard error between two biological replicates, which is equal to one half the range. (TIF) [file pgen.1010898.s002.tif]
