# Supplementary material for: Kar4 is required for the normal pattern of meiotic gene expression
Source: PLoS Genet. 2023 Aug 28;19(8):e1010898. doi: 10.1371/journal.pgen.1010898 (PMC10491391; doi:10.1371/journal.pgen.1010898)
Supplement: S1 Table — All auxotrophic markers are standard BY alleles. (DOCX) [file pgen.1010898.s003.docx]

**S1 Table. Strains used for this study. All auxotrophic markers are standard BY alleles.**

| Strain Name | Genotype | Strain |
| --- | --- | --- |
| MY 10128 | *leu2 his3 ura3 met15 kar4::KANMX* | S288c |
| MY 11297 | *ura3 leu2 his3 lys2 can1::LEU2+-MFA1pr-HIS3 kar4::KANMX* | S288c |
| MY 8092 | *leu2 his3 ura3 met15* | S288c |
| MY 16616 | *his3/" leu2/leu2::pACT1-Z3EV-NATMX +/lys2 met15/+ ura3/"* | S288c |
| MY 16533 | *HPHMX::PZ3EV-IME1/+ HPHMX::PZ3EV-RIM4/+ leu2::act1pr-Z3EV-NATMX/" ura3/"* | S288c |
| MY 16534 | *HPHMX::PZ3EV-IME1/+ leu2::act1pr-Z3EV-NATMX/leu2 ura3/" his3/+ lys2/+* | S288c |
| MY 16617 | *his3/" leu2/leu2::pACT1-Z3EV-NATMX +/lys2 met15/+ ura3/" kar4::KANMX/"* | S288c |
| MY 16531 | *HPHMX::PZ3EV-IME1/+ leu2::act1pr-Z3EV-NATMX/leu2 ura3/" his3/+ met15/+ kar4::KANMX/kar4::URA3* | S288c |
| MY 16536 | *HPHMX::PZ3EV-IME1/+ HPHMX::PZ3EV-RIM4/+ leu2::act1pr-Z3EV-NATMX/" ura3/" kar4::LEU2/kar4::URA3* | S288c |
| MY 16208 | *HPHMX::PZ3EV-IME1/+ leu2::act1pr-Z3EV-NATMX/leu2 ura3/" his3/+ met15/+ kar4::KANMX/+ GAS4-3HA::HIS3/+* | S288c |
| MY 16209 | *HPHMX::PZ3EV-IME1/+ leu2::act1pr-Z3EV-NATMX/leu2 ura3/" his3/+ met15/+ kar4::KANMX/+ SPS2-3HA::HIS3/+* | S288c |
| MY 16210 | *HPHMX::PZ3EV-IME1/+ leu2::act1pr-Z3EV-NATMX/leu2 ura3/" his3/+ met15/+ kar4::KANMX/kar4::URA3 GAS4-3HA::HIS3/+* | S288c |
| MY 16211 | *HPHMX::PZ3EV-IME1/+ leu2::act1pr-Z3EV-NATMX/leu2 ura3/" his3/+ met15/+ kar4::KANMX/kar4::URA3 SPS2-3HA::HIS3/+* | S288c |
| MY 16216 | *HPHMX::PZ3EV-IME1/+ HPHMX::PZ3EV-RIM4/+ leu2::act1pr-Z3EV-NATMX/" ura3/" kar4::KANMX/" GAS4-3HA::HIS3/+* | S288c |
| MY 16217 | *HPHMX::PZ3EV-IME1/+ HPHMX::PZ3EV-RIM4/+ leu2::act1pr-Z3EV-NATMX/" ura3/" kar4::KANMX/" SPS2-3HA::HIS3/+* | S288c |
| MY 16572 | *his3/" leu2/" ura3/" lys2/+ met15/+ IME2-13MYC::KANMX/+* | S288c |
| MY 16573 | *his3/" leu2/" ura3/" lys2/+ met15/+ IME2-13MYC::KANMX/+ kar4::HPHMX/"* | S288c |
| MY 16604 | *IME2-13MYC::KANMX/+ kar4::HPHMX/kar4::URA3 ura3/" his3/+ leu2/leu2::act1pr-Z3EV-NATMX HPHMX::PZ3EV-IME1/+ met15/+* | S288c |
| MY 16608 | *IME2-13MYC::KANMX/+ ura3/" his3/+ leu2/leu2::act1pr-Z3EV-NATMX HPHMX::PZ3EV-IME1/+ met15/+* | S288c |
| MY 16612 | *HPHMX::PZ3EV-IME1/+ HPHMX::PZ3EV-RIM4/+ kar4::URA3/kar4::LEU2 his3/+ leu2/leu2::act1pr-Z3EV-NATMX IME2-13MYC::KANMX/+ ura3/"* | S288c |
| MY  16643 | *HPHMX::PZ3EV-IME1/+ leu2::act1pr-Z3EV-NATMX/leu2 ura3/" his3/+ met15/+ kar4::KANMX/kar4::URA3 mek1::BLEMX/”* | S288c |
| MY  16644 | *HPHMX::PZ3EV-IME1/+ leu2::act1pr-Z3EV-NATMX/leu2 ura3/" his3/+ met15/+ kar4::KANMX/+ mek1::BLEMX/”* | S288c |
