# Supplementary material for: Kar4 is required for the normal pattern of meiotic gene expression
Source: PLoS Genet. 2023 Aug 28;19(8):e1010898. doi: 10.1371/journal.pgen.1010898 (PMC10491391; doi:10.1371/journal.pgen.1010898)
Supplement: S2 Table — (DOCX) [file pgen.1010898.s004.docx]

**S2 Table. Plasmids used in this paper.**

| Strain | Markers | Source |
| --- | --- | --- |
| pMR6895 | *2µ IME1 URA3 Amp* |  |
| pRB3483 | *hphMx-Pz3ev CEN Amp* | 1 |
| pMR6885 | *2µ NDT80 LEU2 Amp* |  |

**References**

1. McIsaac RS, Gibney PA, Chandran SS, Benjamin KR, Botstein D. Synthetic biology tools for programming gene expression without nutritional perturbations in *Saccharomyces cerevisiae*. Nucleic Acids Res. 2014;42(6):e48. doi: 10.1093/nar/gkt1402. PubMed PMID: 24445804; PubMed Central PMCID: PMCPMC3973312.
